# Isolation and Expression Analysis of Novel Silicon Absorption Gene from Roots of Mangrove *(Rhizophora apiculata) via* Suppression Subtractive Hybridization

**DOI:** 10.1155/2014/971985

**Published:** 2014-01-01

**Authors:** Mahbod Sahebi, Mohamed M. Hanafi, Siti Nor Akmar Abdullah, Mohd Y. Rafii, Parisa Azizi, Naghmeh Nejat, Abu Seman Idris

**Affiliations:** ^1^Laboratory of Plantations Crops, Institute of Tropical Agriculture, Universiti Putra Malaysia, 43400 Serdang, Selangor, Malaysia; ^2^Department of Land Management, Faculty of Agriculture, 43400 Serdang, Selangor, Malaysia; ^3^Laboratory of Food Crops, Institute of Tropical Agriculture, Universiti Putra Malaysia, 43400 Serdang, Selangor, Malaysia; ^4^Biological Research Division, GANODROP Unit, Malaysia Palm Oil Board (MPOB), No. 6, Persiaran Institusi, Bandar Baru Bangi, 43000 Kajang, Selangor, Malaysia

## Abstract

Silicon (Si) is the second most abundant element in soil after oxygen. It is not an essential element for plant growth and formation but plays an important role in increasing plant tolerance towards different kinds of abiotic and biotic stresses. The molecular mechanism of Si absorption and accumulation may differ between plants, such as monocotyledons and dicotyledons. Silicon absorption and accumulation in mangrove plants are affected indirectly by some proteins rich in serine and proline amino acids. The expression level of the genes responsible for Si absorption varies in different parts of plants. In this study, Si is mainly observed in the epidermal roots' cell walls of mangrove plants compared to other parts. The present work was carried out to discover further information on Si stress responsive genes in *Rhizophora apiculata*, using the suppression subtractive hybridization technique. To construct the cDNA library, two-month-old seedlings were exposed to 0.5, 1, and 1.5 mM SiO_2_ for 15 hrs and for 1 to 6 days resulting in a total of 360 high quality ESTs gained. Further examination by RT-PCR and real-time qRT-PCR showed the expression of a candidate gene of *serine-rich protein*.

## 1. Introduction

Abiotic stresses, such as drought, high salinity, temperature, chilling, and high intensity light, usually affect higher plants by preventing plant growth. Improvement in biotic and abiotic resistance in crops and trees plays a vital role in both creation of sustainable agriculture systems, through suppression of deleterious effects of global warming *via* decreasing amounts of CO_2_ in the atmosphere, and provision of sufficient food sources in third-world countries. Isolation of resistance-stress genes is an important key to improve stress-susceptibility in plants [1].

Mangrove plants which grow well in plant nutrient poor conditions with high rate of salinity could be a valuable source of antibiotic and abiotic stress genes. Mangrove roots are also able to absorb water from anaerobic soils and in order to maintain the absorbed water the plants need to respire easily which is enabled by their pneumatophores or aerial roots [2].

Mangrove forests have extremely productive ecosystems with an average production of 2,500 mg C cm^−2^ day^−1^ over and above a productivity factor of 4 in the shelf regions to 40 in an open ocean [3–5]. The high rate of organic matter productivity and the external exchange with marine and terrestrial ecosystems* via* biochemical carbon cycling highlights the importance of the mangrove in tropical coasts [6].

Harsh environmental conditions provide for a great deal of physiological and basic adaptations in mangrove plants which consequently allow them to overcome a wide range of abiotic stresses and survive. Wetland sediments created by rivers are significantly unsteady and anaerobic as well as full of sulfates, which lead to pressure on mangrove plants to adapt as far as possible [7].

A few efforts have been made to understand the intraspecific variations of mangroves and to predict the performance of mangrove ecosystems. Mangrove ecosystems have remained almost intact as a widespread gene pool because of lack of regular morphological variations between species and among the populations, although the structure of mangrove species population for many aquatic organisms has been identified [8–11]. The major concern is to find how their genetic structure is organized and to determine the correlation between different traits, which include adaptive and nonadaptive, with migration of diverse genes, which leads to evaluation of developmental changes in mangrove ecological conditions [8]. Mangrove trees are capable of decreasing nutrient losses when there are changes in atmospheric conditions by applying a variety of mechanisms, including biogeochemical and physiological, while exposed to a waterlogged and salty environment [12–14]. Ion preservation, immobilization, and translocation in soaked soil, efficiency of nutrient use, which is the highest recorded among trees, and the morphological shape of its roots probably play an important role in establishing these mechanisms [14].

Among the plant nutrient elements in soil, Si is the most abundant, after oxygen, and essential for plant formation under poor nutrient conditions. The role of Si is not limited to plant growth as it also plays an important role in decreasing the susceptibility of plants to different environmental stresses [15–19]. *Serine- and proline-rich proteins* play a significant role in plants with regard to Si absorption and transportation [20, 21]. In the present study, we isolated and identified *serine-rich protein* genes from the roots of the mangrove plant (*R. apiculata).*

Currently, many methods are being used to study differentials in the expression of genes including serial analysis of gene expression (SAGE), differential display reverse transcription-polymerase chain reaction (DDRT-PCR), cDNA microarray, suppression subtractive hybridization (SSH), and cDNA-AFLP. The false positive created through the SSH technique is much lower compared to that through the other methods [22, 23].

## 2. Materials and Methods

### 2.1. Plant Materials

Mangrove (*Rhizophora apiculata*) seeds were collected from Kuala Sepetang (04° 50.150′′N, 100° 37.620′′) in Taiping, Perak, Malaysia. They were grown in hydroponic culture for two months and then treated with 0.5, 1, and 1.5 mM SiO_2_ for 15 hrs and for 1 to 6 days. The roots of the plants were collected, immediately washed with distilled water, and frozen in liquid nitrogen to facilitate the RNA extraction process.

### 2.2. Total RNA Extraction and Construction of the cDNA Library

The RNA extracted from the roots of the mangrove was isolated using the CTAB method [24]. The quality and integrity of the extracted RNA were assessed using the NanoDrop ND-1000 spectrophotometer (NanoDrop Technologies, USA). Poly(A) + RNA was extracted from total RNA using a PolyATtract mRNA Isolation Kit (Promega, USA).

The subtracted cDNA library was constructed using the PCR-Select Subtractive Hybridization Kit (Clontech, USA), following the manufacturer's instructions. In brief, mRNAs from the last step for both control and treated samples were designated as driver and tester, respectively. The first-strand and double-strand cDNAs were then synthesized and the synthesized double-strand cDNA of the tester sample was digested with restriction enzyme Rsa I. The digested tester cDNA (blunt ends) was divided into two parts which were subsequently ligated with two different kinds of cDNA adaptors (long inverted terminal repeats) A and B. In order to normalize and enrich mangrove root development-related Si absorption genes that are up- or down-regulated by Si stress, two rounds of hybridizations and suppression PCR amplification were processed. The PCR products of secondary PCR amplification were then purified and inserted directly into the pDrive U/A cloning vector (Qiagen, Germany). The ligated pDrive vectors were then transformed into *E.  coli* EZ cells and cultured overnight (16 hrs, 37°C) in LB agar medium containing X-gal, IPTG, and ampicillin. A total of 400 independent positive white clones were picked out randomly, put in LB broth containing Amp, and incubated at 37°C overnight to establish the mangrove root subtractive library.

### 2.3. EST Sequencing and Analysis

About 400 positive clones were selected randomly and amplified using M13 primers (forward and reverse) after removal of contamination from the vector and primer sequence. Before the assembly search, adaptors, polyA tails, low quality sequences, short sequences less than 100 bp in length, and vector sequences were removed. The algorithm search of contigs and singletons was performed using CAP3 software. This was followed by the obtained sequences being submitted to the NCBI database for homology search.

The BLASTn was used to show degree of similarity between the clone cDNA sequence and a known sequence and the BLASTx (http://blast.ncbi.nlm.nih.gov) showed function of qualified cDNA sequences with large ORF regions. Classification of cDNA sequences was based on their *E*-value results in the BLAST. Categories of sequence functions are based on the Blast2GO program (http://www.blast2go.org/) [25].

Computational annotation of the mangrove EST datasets was performed using the Blast2GO software v1.3.3 (http://www.blast2go.org/). The BLAST search was performed at NCBI [26]. The degree of amino acid sequence similarity was determined by the use of Wu-Blast from EBI [27]. Hydrophilicity and hydrophobicity of *serine-rich protein* were predicted online by MemBrain, TMHMM, and ProtScale (http://web.expasy.org/protscale/) in the toolkit of ExPASy. Subcellular localization was investigated using PSORT II Prediction and Cell-PLoc, BaCelLo program. The prediction of secondary structure was carried out by (http://npsa-pbil.ibcp.fr/cgi-bin/npsa_automat.pl?page=nps a_sopma.html) and PsiPred program. The prediction of 3D structure was carried out by using the Pfam program.

### 2.4. Amplification of Full-Length cDNA

The complete CDS of *serine-rich protein* gene contained 696 bp and 223 amino acids. The PCR program according to KAPA HiFi Hot Start was used as follows: initial denaturation at 95°C for 5 min, 35 cycles of denaturation at 98°C for 30 s, annealing at 57.5°C for 30 s, and extension at 72°C for 1 min. The final extension was 5 min at 72°C. Agarose gel (1.5%) was used to separate PCR-amplified cDNA fragments. The expected bound about 700 bp was purified using gel purification kit (Qiagen, Germany) and 3′-dA-overhangs (incubation 72°C for 5 min) added to the blunt-ended DNA fragments generated by KAPA HiFi Hot Start DNA polymerase to ligate into the pDrive cloning vector (Qiagen, Germany) and sent for sequencing.

### 2.5. Semiquantitative RT-PCR Analysis

Reverse transcriptase RT-PCR was performed to study the expression of the serine-rich protein gene. One *μ*L of DNase treated (DNase I, Qiagen, Germany) total RNA from each of the mangrove roots treated with Si for 15 hrs and 1 to 6 days and untreated plants was transcribed to the first-strand cDNA using SuperScript III (Invitrogen, USA) and 500 ng oligo (dT) 18 primer in 20 *μ*L reaction volume. Reactions were then incubated at 50°C for 60 min and heated to inactive at 70°C for 15 min. The template cDNAs for both control (untreated) and treated samples were then amplified using serine primers as F: 5-GTCATTCTGCCGAGTTCC-3 and R: 5-AATGCCCATTTATGTGACTTCG-3 designed according to the cDNA sequence homology.

Actin gene as an internal control was amplified with the following primers:F: (5′CAC TAC TAC TGC TAA ACG GG AAA 3′) and R: (5′ACA TCT GCT GGA AGG TGC TG 3′). The following PCR (Tag DNA Polymerase, Vivantis, USA) program was used: 94°C for 2 min and 35 cycles of 94°C for 30 sec, 57.5 and 58°C, respectively, for actin and serine for 30 sec, and 72°C for 30 sec. The PCR program was concluded with final extension of 7 min at 72°C. Actin and *serine-rich protein* were amplified using the same cDNA templates. The PCR products then were separated with 1.5% agarose gel and stained with ethidium bromide.

### 2.6. Real-Time Quantitative RT-PCR Analysis

Real-time qRT-PCR was performed to evaluate the expression levels of candidate *serine-rich protein* genes in the root tissues in response to treatments with different concentrations of Si compared to these in the control plants. Total RNA was extracted from untreated and Si-treated mangrove roots. Extracted RNA was then treated with DNase (DNase I, Qiagen, Germany). The primers for the actin gene were used as in the previous section and for the second endogenous control ef*α*1 was F 5′→ 3′ ATT GGA AAC GGA TAT GCT CCA R 5′→ 3′ TCC TTA CCT GAA CGC CTG TCA; *serine-rich protein* gene primer was F: 5′→3′GCAAGTGCTATGTTCAGGCA, R: 5′→3′AACAATAACGATGGCAAGGC. One *μ*L aliquot of DNase treated RNA from each sample was used to prepare 20 *μ*L reaction volumes (based on the KAPA SYBER FAST One-Step qRT-PCR). The reactions involved were a primary incubation of 42°C for 5 min, inactive RT at 95°C for 5 min, followed by 40 cycles at 95°C for 3 sec, 60°C for 30 sec, and 72°C for 3 sec. The 96-well plate was used to analyze each sample in duplicate. In this study all data was from four independent biological replicates. A standard curve (*R* ^2^ > 0.95) from 10-fold serial dilutions was generated from the purified cDNA fragments of internal and *serine-rich protein* genes (10^2^, 10, 1, 10^−1^, and 10^−2 ^ng/*μ*L).

## 3. Results

### 3.1. Yield and Integrity of RNA

The RNA appeared as a nondegraded band on 1.5% agarose gel containing formaldehyde. The A_260/280_ ratios ranging from 1.9 to 2.02 indicate that there was no protein contamination and the A_260/230_ ratio >1 demonstrated that there is no polyphenol or polysaccharide contamination [28]. The RNA concentration was in the range of 0.5–1.2 mg g^−1^. The poly(A) + RNA of both treated and untreated samples appeared as clear smears on the 1% agarose gel with the A_260/280_ ratio = 2 indicating high quality of mRNA obtained for further analysis.

### 3.2. Construction of the Subtracted cDNA Library

The products of the subtracted suppression hybridization cDNA library appeared on 1.2% agarose gel as a smear with ranking size from 150 bp to 1.2 kb and 4–6 separate bands (Figure 14, line A) which obviously differentiates them from the unsubtracted sample driver (Figure 14, line B). The results of the SSH cDNA library indicated that the differentially expressed genes are present in the tester or treated samples and absent or present at lower levels in the driver or untreated samples. For further confirmation of subtraction analysis efficiency, expression of the actin gene was examined in both the driver and control samples *via* 23 and 33 cycles of amplification, respectively, indicating that cDNA homologue was removed from both the tester and driver samples by subtraction (Figure 14).

### 3.3. ESTs Sequencing and Gene Annotation

**Figure 1:**
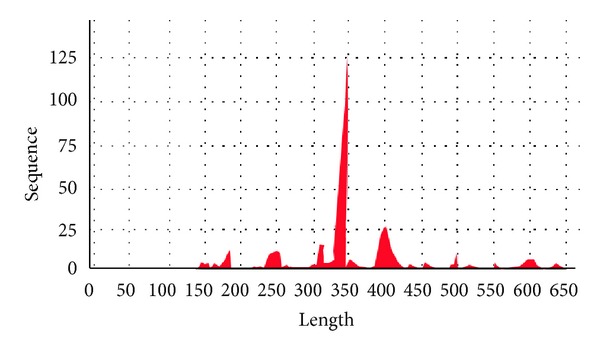
Number of sequences with length which resulted from the subtracted cDNA library.

**Figure 2:**
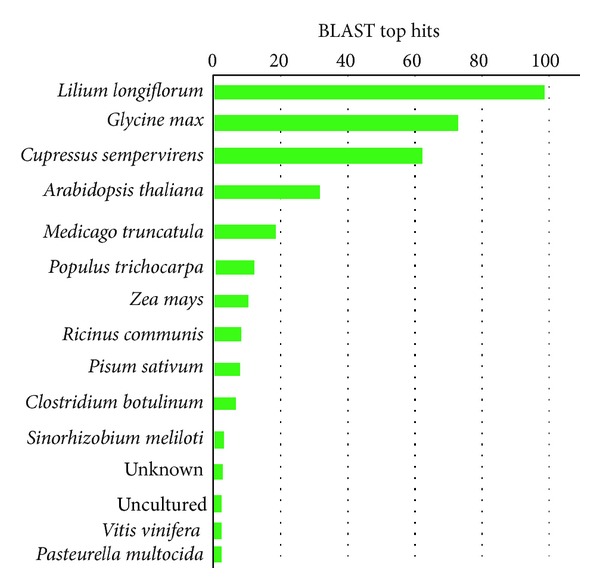
Top hit distribution of ESTs analysis.

About 700 positive recombinant clones were isolated from the cDNA library. Of those, about 400 clones were randomly selected, sequenced, and analyzed to isolate the gene(s) involved in Si transportation and absorption. The 322 ESTs sequences were coalesced into 21 contigs and 13 singletons by CAP3 assembly program. About 19.5% of the ESTs resulting from this library did not have any significant homology to any of the proteins existing in the database. The DNA fragments involved in the subtracted cDNA library varied in size, more or less between 100 and 650 bp (Figure 1). Average length of high quality ESTs is 350 bp. The most plentiful BLASTx hits correlated to species distribution related to *Lilium longiflorum, Glycine max, Cupressus sempervirens, and Arabidopsis thaliana* (Table 1 and Figure 2).

### 3.4. Classification of Differentially Expressed Genes

**Figure 3:**
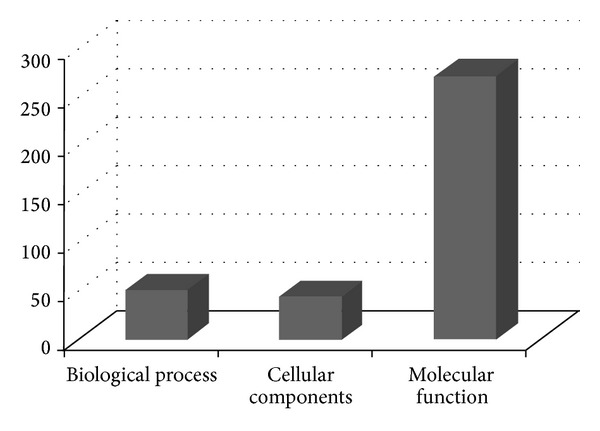
Gene annotation of 322 ESTs which resulted from the SSH library.

**Figure 4:**
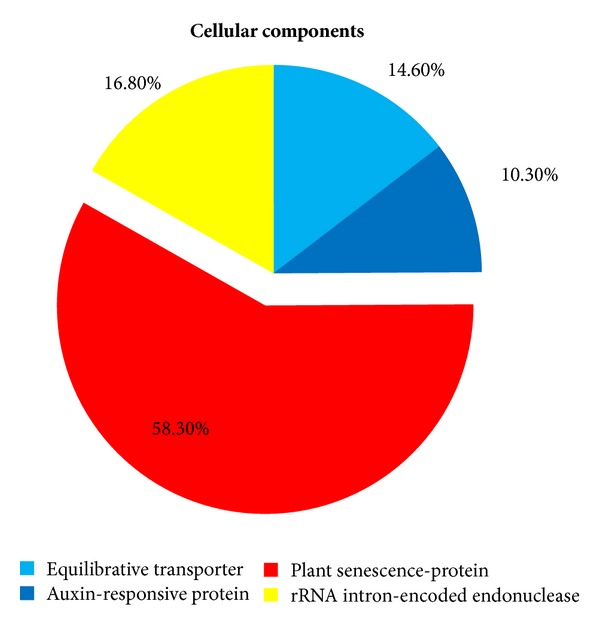
Cellular components categorization of cDNA library result.

**Figure 5:**
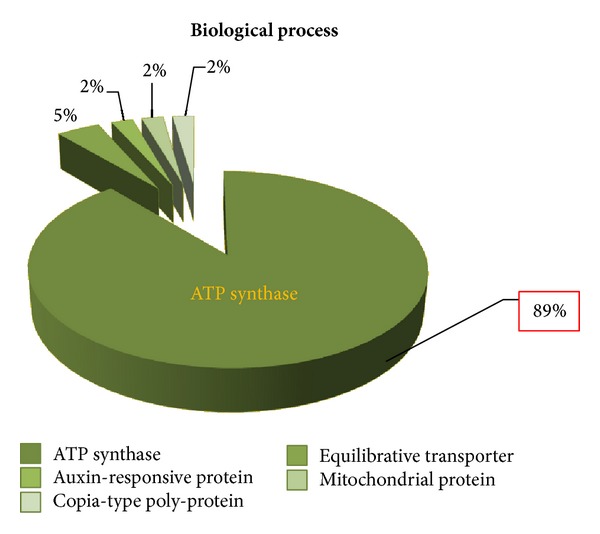
Biological process categorization of the subtracted cDNA library.

**Figure 6:**
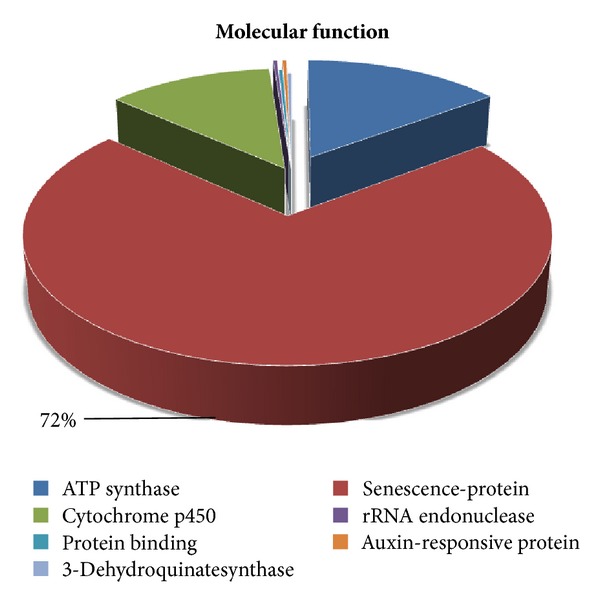
Molecular function categorization of the subtracted cDNA library.

According to gene annotation, the 322 ESTs were divided into three different groups involving biological process, molecular function, and cellular components (Figure 3). The potential identities of the genes are based on similarities to those present in the Gen-Bank databases. The genes obtained by the SSH library classified by biological process involve 5 different groups: ATP synthase (88.9%), equilibrative transporter (4.4%), auxin-responsive protein (2.2%), mitochondrial protein (2.2%), and copia-type polyprotein (2.2%); those classified according to cellular components involve 3 different groups. The ATP synthase was highly abundant in both the cellular components and biological process categories (Figures 4 and 5), while senescence-protein was the most abundant group in the molecular function category (Figure 6). Classification of the ESTs resulted in 94% of known, 4% hypothetical, and 2% unknown functions.

### 3.5. Isolation of the Full-Length Serine-Rich Protein Gene

**Figure 7:**
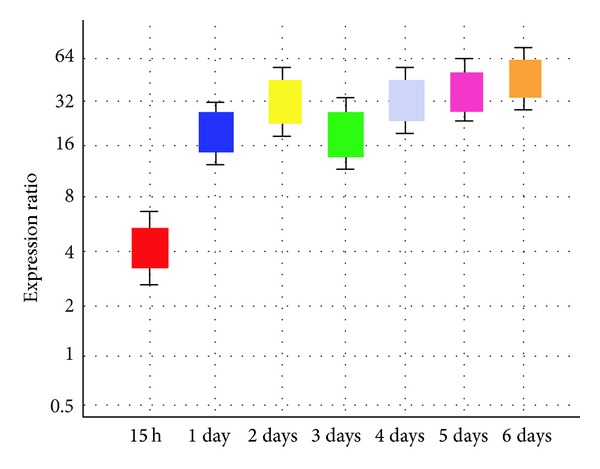
Relative quantity of *serine-rich protein* gene and actin as an internal control. Serine 15 h sample group is not different to control group. *P* value = 0.169. Serine 1 day is up the regulated in sample group (in comparison to control group). *P* value = 0.000. Serine 2 days is up-regulated in sample group (in comparison to control group). *P* value = 0.000. Serine 3 days sample group is not different to control group. *P* value = 0.339. Serine 4 days is up-regulated in sample group (in comparison to control group). *P* value = 0.000. Serine 5 days is up-regulated in sample group (in comparison to control group). *P* value = 0.000 Serine 6 days is up-regulated in sample group (in comparison to control group). *P* value = 0.000.

**Figure 8:**
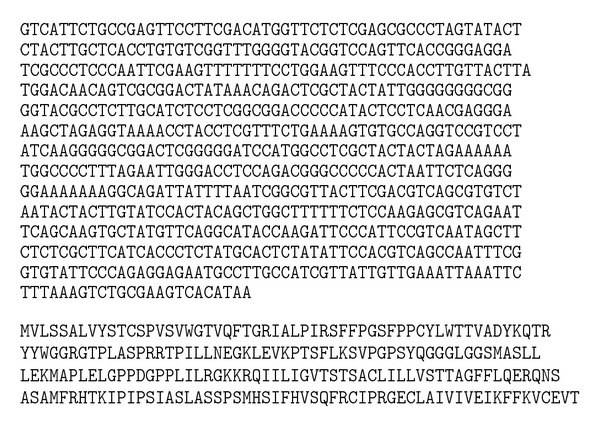
The nucleotide (696 bp) and deduced amino sequence (223 aa) of *serine-rich protein*.

One of the ESTs sequence showing 97% similarity involves the ATG codon (20% query cover region) with complete CDS of the *serine-rich protein* gene of *Arachis hypogaea* (Figure 7). Full length of the gene (Figure 8) was obtained through amplification of the cDNA template (First-Strand cDNA synthesis, Invitrogen, USA) and using gene-specific primers as follows:

 serine
 F: 5′ → 3′ GTCATTCTGCCGAGTTCC
 R: 5′ → 3′AATGCCCATTTATGTGACTTCG.

### 3.6. Analysis of the Differential Expression of Serine-Rich Protein Gene Using Semi-qRT-PCR and Real-Time qRT-PCR

The semiquantitative RT-PCR analysis showed that the expression levels of *serine-rich protein* were generally higher in the Si treatment samples than in the untreated samples. The expression level of *serine-rich protein* in the 3-day treated sample was lower compared to that in the other treated samples with varying periods of time (Figure 15). Real-time qRT-PCR confirmed the results of the semi quantitative RT-PCR (Figure 8). The relative transcript abundance of *serine-rich protein* in Si-treated plants was higher compared to that in the untreated plants.

The PCR efficiency of all reactions in this study was between 87 and 98%. The REST software (Qiagen, Hilden, Germany) was employed to analyze the results of the qRT-PCR. The manufacturer's instructions were followed to quantify the relative gene expression. Differences among treatment samples were noted as being statistically significant (*P* < 0.05).

### 3.7. Bioinformatics Analysis

The low quality regions at the end and beginning of each sequence were trimmed using a Phred 20 cutoff value. Vector screening was carried out using the crossmatch. Oligo dT tracks and other contaminants were removed. Algorithms of CAP3 [29] assembly were used to assemble the individual ESTs into clusters of sequences derived from the same transcript as tentative consensus sequences (TCs) and singletons representing unique transcripts. Obtained sequences were submitted to NCBI database.

**Figure 9:**
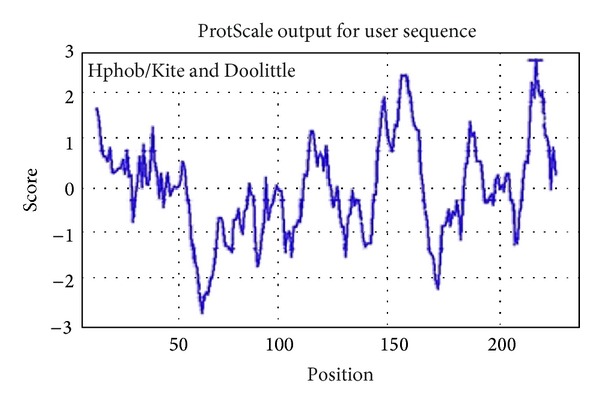
Analysis of hydrophilicity and hydrophobicity for the *serine-rich protein*. This figure is the ProtScale output of hydrophilicity and hydrophobicity for the *serine-rich protein*.

**Figure 10:**
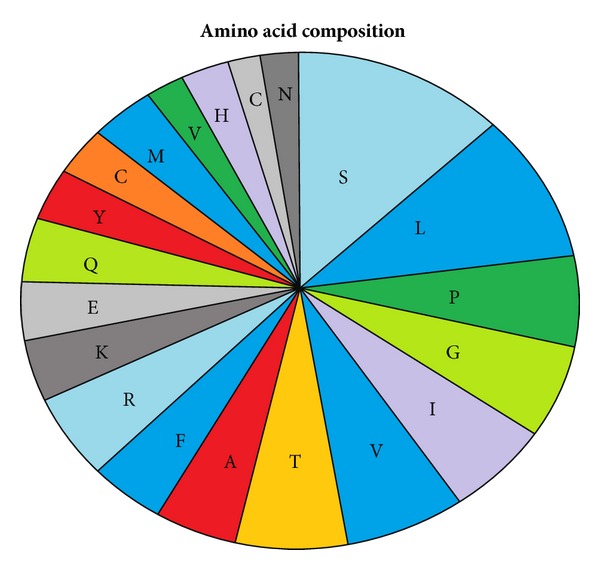
Amino acid composition of *serine-rich protein*.

Prediction of hydrophilicity and hydrophobicity is needed for predicting protein secondary structure and division of functional domain, according to the theory that hydrophilicity and hydrophobicity are related to the score of amino acid. Based on the number of hydrophilic amino acid residues, we could hypothesize that the *serine-rich protein* was a hydrophilic protein (Figures 9 and 10).

### 3.8. The Prediction of Secondary Structure and Function Domain of Serine-Rich Protein

**Figure 11:**
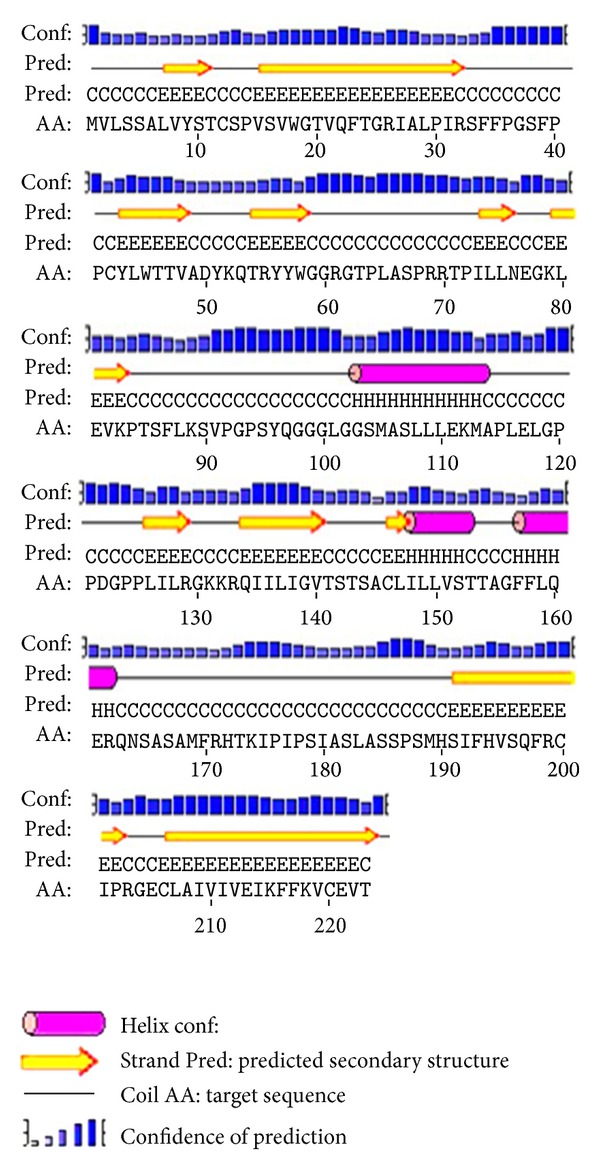
Prediction of secondary structure for the *serine-rich protein*.

The prediction results of secondary structure of *serine-rich protein* by PsiPred showed that the secondary structure consisted of 11 sheets, 3 helixes, and 15 coils (Figure 11). Computational analysis of the cDNA clone isolated from mangrove root library indicated that its 696 bp coding region codes for a protein of 223 amino acids with a predicted molecular mass of 24.21 kDa. Homology searches run with the full-length amino acid sequences.

### 3.9. Subcellular Localization

**Figure 12:**
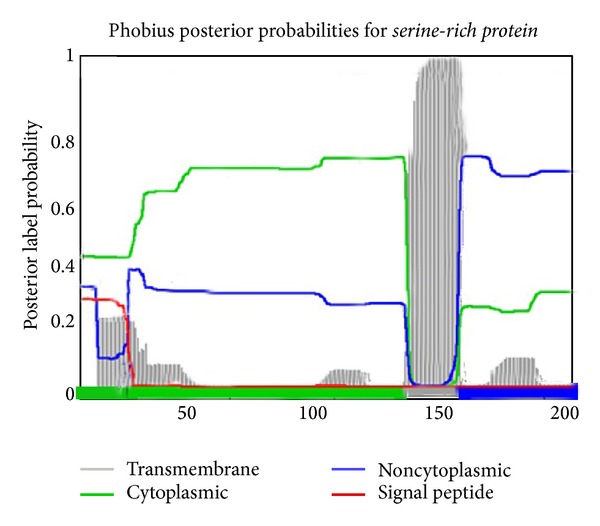
Prediction of subcellular location of *serine-rich protein*.

The prediction of subcellular localization with Cell-PLoc, BaCelLo, and WoLF PSORT showed that *serine-rich protein* is likely to be localized in chloroplast, plastid, and mitochondrion, with a different probability of 17, 5, and 1%, respectively (Figure 12). Biosequence analysis of coding sequence region (CDS) of serine-rich protein using profile hidden Markov models (HMMER) showed 60% similarity to *serine-rich protein* of *Arachis hypogaea* (TR:Q0MX20_ARAHY) and 100% similarity to mitochondrial protein of *Medicago truncatula* (TR:G7I9T8_MEDTR) (Table 2).

### 3.10. Prediction of 3D Structure

**Figure 13:**
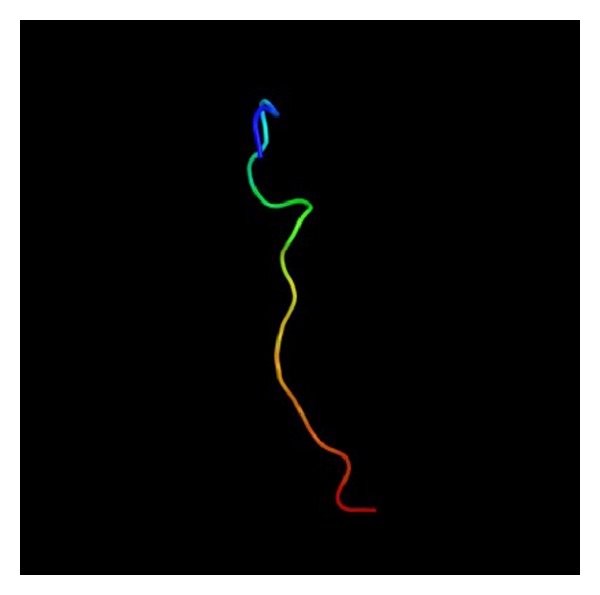
3D structure of serine-rich protein. The protein folds are shown in the colors of the rainbow from the N terminus (blue) to the C terminus (red).

**Figure 14:**
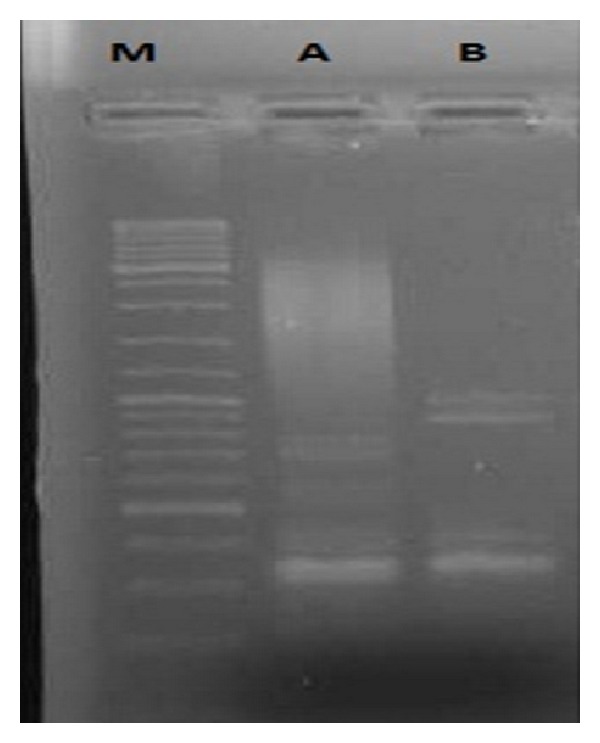
Result of the SSH cDNA library. Lane M: marker; lane A: subtracted driver sample after second PCR; lane B: subtracted tester sample after second PCR.

In order to predict the 3D structure of *serine-rich protein*, the Phyre2 (Protein Homology Analogy Recognition Engine) server was used [30]. A Phyre2 output model was generated based on the template galactose-binding domain-like. Structural alignment of *serine-rich protein* and galactose-binding domain-like was performed using the Matchmaker tool of UCSF Chimera [31]. The 3D structure protein of *serine-rich protein* gene isolated and identified in the present study submitted to NCBI with accession number KF211374 involves MYP, b/Zip, LCR1, and DOF transcription factor binding motifs (Figure 13).

## 4. Discussion

In the present study, the suppression subtractive hybridization (SSH) method was used to identify differentially expressed genes present in the tester sample and absent or present at lower levels in the driver. The SSH cDNA library was constructed using SiO_2_-treated (15 hrs and 1 to 6 days) roots of mangrove plants, whereby 321 unique genes were obtained. The SSH method provides information related only to global analysis of gene expression, and hence semi quantitative RT-PCR and real-time qRT-PCR were performed to evaluate the quantitative level of gene expressions over varying periods of Si treatment. Most sequences recognized from this subtracted library encoded different genes with homology of genes from 13 known and 2 unknown species.

**Figure 15:**
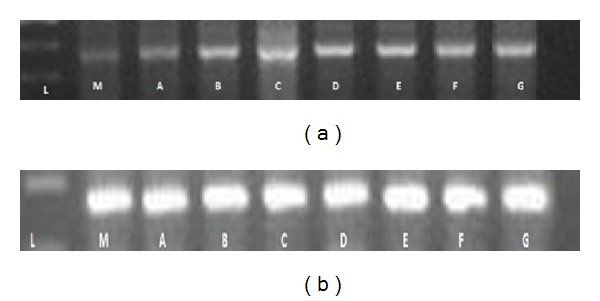
Relative expression of *serine-rich protein* gene (a) and actin as an internal control (b) was amplified by semi-qRT-PCR. L: molecular ladder, M: untreated plants, A: 15 hrs silicon treated, B: 1-day silicon treated, C: 2-days silicon treated, D: 3-day silicon treated, E: 4-day silicon treated, F: 5-day silicon treated, and G: 6-day silicon treated.

One EST with known function was selected for analysis of Si-induced expression patterns in varied periods of time using semi quantitative reverse transcriptase and real-time qRT-PCR (Figures 15 and 7).

The SSH cDNA library of Si-treated genes was monitored for seven times. From SSH reports, it appears that some gene induction patterns are so rapid and self-motivated that they may not be detected by Si stress applied. The results of the SSH library constructed in this study contain the majority of induced genes, such as serine-rich protein which was upregulated and recovered from the roots of two-month old mangrove seeds after Si treatment. Previous studies identified *Lsi1*, *Lsi2*, and *Lsi6* as the genes responsible for Si transportation and accumulation, isolated from roots and leaves of rice and corn. The capability of an influx transporter on one side and an efflux transporter on the other side of the cell to permit effective transcellular transport of the nutrients was revealed after identification of *Lsi1* and *Lsi2*.

The protein encoded by these genes is localized, like *Lsi1*, on the plasma membrane of cells in both the exodermis and the endodermis. However, in contrast to *Lsi1*, which is localized on the distal side, *Lsi2* is localized on the proximal side of the same cells [32]. Meanwhile, Yamaji et al. [33] reported on the role of *Lsi6* in plant nutrient redirection at the node I part. A corollary to Yamaji's [33] result is that the role of *Lsi6* could be as a transporter involved in intravascular Si transportation. In the present study, it is reported that the *serine-rich protein* could have a similar role in mangrove roots.

The presence of a trichloroethyl-ester-protecting group and an alcoholic-hydroxyl-group-protecting group in serine is easy to strip down [20] when subjected to modification with higher Si content. Furthermore, serine derivatives can selectively take part in a plurality of chemical reactions and generate derivatives that can perform multiple derivatization reactions [20]. Apart from *serine-rich proteins* some other glycoproteins and polysaccharides may have roles in Si transportation and accumulation. For instance, Kauss et al. [21] argued that the results of Si deposition did not follow an equal independency from accumulation of phenolics in every plant. Moreover, Perumalla and Heath [34] mentioned that 2,2′-dipyridl, an iron-chelating inhibitor of proline hydroxylation, decreased the number of Si deposition sites. Hence, there is a relationship between availability of proline and deposition of Si in plants.

Mangroves in association with Si may involve many glycoproteins and polysaccharides enriched by many amino acids, including threonine, proline, serine, glycine, glutamic acid and aspartic acids, which are OH-terminated. The SSH library was successful in identifying 4 ESTs associated with the *serine-rich protein* mRNA, the sequence submitted to the Gen-Bank (Access. number DQ834690.1), and 2 ESTs associated with *proline-rich proteins*, the sequence submitted to the Gen-Bank (Access. number AAB70928.1). It has been reported that these polysaccharides play an important role as stable intermediates in Si accumulation, transportation, and nucleation [20, 21].

Many ESTs associated with transcription regulatory and signal transduction were isolated from the Si-induced mangrove SSH library, including ATP synthase subunit beta, mitochondrial protein, and auxin-responsive protein.

Several ESTs isolated were homologous to senescence-associated proteins. Senescence-associated protein functions as a defense mechanism in response to diseases caused by fungi, bacteria, and viruses [35]. Several ESTs isolated from the SSH library were homologous to cytochrome p450-like tbp protein involved in molecular functions, such as hydroxylation and molecular trajectories for hydroxylation [36]. The other less abundant ESTs identified by the SSH library involved one EST, which was homologous to the equilibrative nucleoside transporter (Gen-Bank Access. number XP_002317251.1); four ESTs associated with rRNA intron-encoded endonuclease (Gen-Bank Access. number BAD18905.1); one EST homologous to protein binding protein, putative (Gen-Bank Access. number XP_002515352.1); one EST associated with 3-dehydroquinate synthase, putative (Gen-Bank Access. number XM_003588307.1); one EST homologous to mitochondrial protein, putative (Gen-Bank Access. number AES58606.1); and one EST homologous to copia-type polyprotein (Gen-Bank Access. number CAB71063.1).

The analysis and EST data presented here are a first global overview of Si absorption genes in mangrove. The annotation of these ESTs has identified many genes associated with or having a potential role in Si absorption. These genes provide a starting point for understanding the nature of molecular mechanisms of plant's Si absorption. Energy conversion processes of the protein of interested gene are involved in increasing the amount of some amino acids, such as serine and glutamic acid, as we observed in the other study which has been done over a functional study of the transgenic *Arabidopsis thaliana* using the HPLC method. On the other hand it has been reported that the biosilica formation in different organisms is under control by increase in the amount of some amino acids, such as serine and proline. Hence, we concluded that transformation of *serine-rich protein* can be effective in Si absorption and accumulation.

In conclusion most of Si-induced SSH mangrove roots were observed to have a putative or known function, whereas some of the ESTs isolated from the SSH have never been recorded from other plant species. Unrecorded genes may have different functions that play different roles in plants and understanding of their functions would be useful in identifying the mechanisms involved in plant breeding and development. In the next phase of experiments the full-length copy of the *serine-rich protein* will be cloned following transgenic *Arabidopsis thaliana* and its function as Si transporter gene analyzed.

## Figures and Tables

**Table 1:** Putative identities of novel silicon-induced cDNA sequences expressed in *R. apiculata* roots.

Gen-Bank accession	Homology	Organism	*E*-value
Stress response			
NP_199564.1	Putative auxin-responsive protein	*Arabidopsis thaliana *	5.00*E* − 19
DQ834690.1	Serine-rich protein mRNA	*Arachis hypogaea *	1.00*E* − 72
AAB70928.1	Proline-rich protein	*Santalum album *	7.00*E* − 11

Transporter			
AET02208.1	ATP synthase subunit beta	*Medicago truncatula *	3.00*E* − 44
XP_002317251.1	Equilibrative nucleoside transporter	*Populus trichocarpa *	7.00*E* − 40

Unknown			
XP_003614394.1	Hypothetical protein MTR_5g051130	*Medicago truncatula *	1.00*E* − 38
XP_003547129.1	Predicted: uncharacterized protein LOC100801	*Glycine max *	6.00*E* − 36
XP_002269167.2	Predicted: uncharacterized protein LOC100267	*Vitis vinifera *	1.00*E* − 30
XP_003549007.1	Uncharacterized protein LOC100791	*Glycine max *	5.00*E* − 43
XP_003541219.1	Uncharacterized protein LOC100796	*Glycine max *	5.00*E* − 43
XP_003541206.1	Predicted: uncharacterized protein LOC100788	*Glycine max *	5.00*E* − 43

Cellular metabolism			
XP_003151440.1	Senescence-associated protein	*Loa loa *	8.00*E* − 25
ACA04850.1	Senescence-associated protein	*Picea abies *	3.00*E* − 21
XP_002139698.1	Senescence-associated protein	*Cryptosporidium hominis TU502*	2.00*E* − 19
AEM36070.1	Senescence-associated protein	*Mytilus eduls *	2.00*E* − 18
EFW13181.1	Senescence-associated protein	*Coccidioides posadasii str*	7.00*E* − 18
XP_002118266.1	Senescence-associated protein	*Trichoplax adhaerens *	1.00*E* − 17
XP_001610563.1	Senescence-associated protein	*Babesia bovis T2Bo*	1.00*E* − 16
XP_003188640.1	Plant senescence-associated protein	*Aspergillus niger CBS 513.88*	5.00*E* − 16
EFN58441.1	Senescence-associated protein	*Chlorella variabilis *	2.00*E* − 11
ACJ09634.1	Putative senescence-associated protein	*Cupressus sempervirens *	4.00*E* − 10
XP_003064992.1	Senescence-associated protein	*Micromonas pusilla CCMP1545 *	1.00*E* − 09
EEH16721.1	Senescence-associated protein	*Paracoccidioides brasiliensis *	2.00*E* − 09
XP_729762.1	Senescence-associated protein	*Plasmodium yoelii yoelii *	2.00*E* − 06
ABO20851.1	Putative senescence-associated protein	*Lilium longiflorum *	1.00*E* − 41
BAB33421.1	Putative senescence-associated protein	*Pisum sativum *	9.00*E* − 17
ACJ09634.1	Putative senescence-associated protein	*Cupressus sempervirens *	1.00*E* − 15
BAD18905.1	rRNA intron-encoded endonuclease	*Thermoproteus *sp. IC-061	6.00*E* − 06
XP_002515352.1	Protein binding protein, putative	*Ricinus communis *	3.00*E* − 34
XM_003588307.1	3-Dehydroquinate synthase, putative	*Medicago truncatula *	5.00*E* − 39
AES58606.1	Mitochondrial protein, putative	*Medicago truncatula *	3.00*E* − 37
CAB71063.1	Copia-type polyprotein	*Arabidopsis thaliana *	3.00*E* − 13
AT4g15300	Cytochrome p450-like tbp protein	*Arabidopsis thaliana *	2.00*E* − 27

**Table 2:** Pfam domain search option for genomic and proteic annotation.

Target	Description	Species	*E*-value
Q0MX20_ARAHY	*Serine-rich protein *	*Arachis hypogaea *	6.80*E* − 147
I3S491_LOTJA	Uncharacterized protein	*Lotus japonicus *	4.90*E* − 05
G7I9T8_MEDTR	Mitochondrial protein, putative (gene: *MTR_1g006300*)	*Medicago truncatula *	0.00084
H6SIC7_RHOPH	Uncharacterized protein (fragment) (gene: *RSPPHO_03263*)	*Rhodospirillum photometricum DSM 122*	0.063
H6SIE2_RHOPH	Uncharacterized protein (fragment) (gene: *RSPPHO_03278*)	*Rhodospirillum photometricum DSM 122*	0.066
H6SID9_RHOPH	Uncharacterized protein (fragment) (gene: *RSPPHO_03275*)	*Rhodospirillum photometricum DSM 122*	0.08
E0XV41_9GAMM	Putative uncharacterized protein	Uncultured Chromatiales bacterium*HF0200_41F04 *	0.098
J2TU55_9PSED	Putative transcriptional regulator (gene: *PMI33_05795*)	*Pseudomonas *sp*. GM67 *	0.26
K9NMI2_9PSED	XRE family transcriptional regulator (gene: *PputUW4_03245*)	*Pseudomonas *sp.*UW4 *	0.27
J3FPP9_9PSED	Putative transcriptional regulator (gene: *PMI26_03923*)	*Pseudomonas *sp*. GM33 *	0.27
J2TNB7_9PSED	Putative transcriptional regulator with cupin domain-containing protein (gene: *PMI34_05211*)	*Pseudomonas *sp.*GM74 *	0.27
J2U6Q2_9PSED	Putative transcriptional regulator (gene: *PMI32_02733*)	*Pseudomonas *sp.*GM60 *	0.28
J3GM97_9PSED	Putative transcriptional regulator with cupin domain-containing protein (gene: *PMI31_04761*)	*Pseudomonas *sp*. GM55 *	0.33
J2SDC1_9PSED	Putative transcriptional regulator (gene: *PMI29_04231*)	*Pseudomonas *sp.* GM49 *	0.45
J3GDW6_9PSED	Putative transcriptional regulator with cupin domain (gene: *PMI28_00614*)	*Pseudomonas *sp*. GM48 *	0.45
D2YVG0_VIBMI	Putative uncharacterized protein (gene: *VMD_37440*)	*Vibrio mimicus VM573*	0.57
B1TDE7_9BURK	Cupin 2 conserved barrel domain protein (gene: *BamMEX5DRAFT_5813*)	*Burkholderia ambifaria MEX-5*	0.64
Q1BPU7_BURCA	Transcriptional regulator, XRE family (gene: *Bcen_3464*)	*Burkholderia cenocepacia *(strain*AU 1054*)	0.95
B1K857_BURCC	Transcriptional regulator, XRE family (gene: *Bcenmc03_5384*)	*Burkholderia cenocepacia *(strain*MC0-3*)	0.95
D1RK09_LEGLO	Putative uncharacterized protein (gene: *LLB_2711*)	*Legionella longbeachae D-4968*	0.98
